# Usefulness of Biomarkers in Work-Related Airway Disease

**DOI:** 10.1007/s40521-017-0121-9

**Published:** 2017-05-11

**Authors:** Agnieszka Lipińska-Ojrzanowska, Andrzej Marcinkiewicz, Jolanta Walusiak-Skorupa

**Affiliations:** Department of Occupational Diseases and Environmental Health, Nofer Institute of Occupational Medicine, 8 St. Teresy, 91-348 Lodz, Poland

**Keywords:** Biomarkers, Airway inflammation markers, Work-related respiratory diseases, Work-related airway diseases, Work-related respiratory disorders, Work-related airway disorders occupational allergy, Occupational inhalant exposure

## Abstract

Determination of biomarkers may be useful in the surveillance of occupational exposure and workers’ health. The possibility of predicting development/clinical course of specific disorders or current disease, diagnosing in early steps, and health condition monitoring is a real necessity. Various agents present in the workplace environment (or their metabolites) can be measured in samples possessed from human body (blood and urine, saliva, etc.). On the other hand, inhalant exposure may induce specific or non-specific, local or systemic, acute or chronic biological response expressed by synthesis or releasing specific or non-specific substances/mediators that also can be determined in blood, nasal and bronchial lavage or sputum, tear fluid, exhaled breath, etc. The least is known about genetic markers which may predict individual susceptibility to develop some work-related disorders under the influence of occupational exposure. Due to common exposure to inhalant agents at workplace, researches on biomarkers that allow to inspect the impact of exposure to humans’ health are still needed. The authors of this article summarize the utility of biomarkers’ determination in work-related airway diseases in a recent clinical approach.

## Introduction

Work-related respiratory diseases (WRRD) make up to 70% causes of all occupational disorder mortality among working population [1]. In general, classification of WRRD mostly includes interstitial lung diseases (various pneumoconioses and occupational hypersensitivity pneumonitis) and airway diseases (work-related rhinitis, asthma, eosinophilic bronchitis, chronic obstructive pulmonary disease) [1]. Occupational lung cancers are usually discussed separately. There is no doubt about the influence of inhalant occupational exposure on workers’ health. Dusts and specific agents may induce or moderate the clinical course of airway disease with both allergic and non-allergic etiologies. Biomarkers are measureable indicators of human exposure to some agents or expressing health condition. Genetic biomarkers may be predictors of individual susceptibility to develop work-related disorders, and their determination could be helpful in typing phenotypes and concluding about illness’ clinical course in the future. Establishment of biomarker significance in work-related airway disorders may be useful in diagnostic process, medical certification of occupational diseases, health monitoring, and even implementation of target therapy.

This article presents reports on biomarkers in work-related airway diseases based on overview of available publications in the EBSCO and the MEDLINE online research databases. The search was limited to articles published mainly in the period of 2005–2016; however, a few previous source publications have been mentioned in references too. Biomarkers that can be determined in biological material samples collected directly from airways seem to be the most useful in diagnostic process and workers’ health monitoring, anywise blood investigation may be applicable too. The conception of “unified airway disease” (UAD) is worth noticing [2, 3] because a lot of biomarkers may be measured in one-type sample during diagnosing or monitoring biological conditions of upper and/or lower respiratory tract.

## Upper and lower airway samples

Investigation on biochemical and cellular composition of nasal lavage fluid (NLF), induced sputum (IS), or bronchoalveolar lavage (BAL) has provided the most reliable outcomes about local inflammatory process in respiratory tract. Additional good point is a non-invasive way of collecting these samples; however, procedures need to be carried out in referential medical centers with appropriate laboratory equipment and qualified personnel. Confirmation of eosinophilic inflammation in nasal secretion samples (smear or lavage) after the challenge with allergens has become the most useful diagnostic phenomenon in occupational rhinitis (OR) [4–7, 8•]. Due to the conception of UAD, increase in the percentage of eosinophils in NLF may be indirectly helpful also in diagnosing occupational asthma (OA), including cough-variant asthma (Corrao’s syndrome) [9]. Although currently the most useful diagnostic tool in OA has become the analysis of biochemical and cytological changes in IS or BAL samples collected pre- and post-challenge work-shift or test conducted in clinical-controlled workplace conditions [10–13]. Confirmation of elevated eosinophil percentage above 3% in IS after specific inhalation challenge (SIC) in comparison to baseline values has been considered to have the highest predictive value for diagnosis of OA [14, 15•]. The development of bronchial eosinophilic inflammation in IS has been observed in sensitized asthmatic patients after SIC with high- (HMW-A) and low-molecular weight allergens (LMW-A) as well [13, 16]. However, establishment of a certain OA recognition demands application of additional diagnostic procedures (e.g., lung function assays) because elevated level of eosinophils in IS has been observed also in solely existing allergic rhinitis, eosinophilic bronchitis (EB), or chronic obstructive pulmonary disease (COPD) [17, 18•].

Alternating biomarker of eosinophilic inflammation in upper and lower airway is eosinophil cationic protein (ECP). Significantly elevated ECP levels have been measured in NLF collected from patients with OR and/or OA [19, 20], also in IS collected from the subjects with OA in the next day after SIC in comparison to baseline assessment [16, 21].

The usefulness of measuring concentration of FE_NO_ in clinical practice is valuable in airway diseases with eosinophilic inflammatory background, especially in asthma and EB. This is not a specific method for work-related respiratory disorders; however, the assessment carried out before and after work-shift or SIC brings information about possible escalation of local inflammatory process, especially if contraindications to induce sputum were certified or the procedure did not allow to obtain sufficient-quality samples [22•, 23]. The measurements of fractional nitric oxide (NO) in exhaled breath have been standardized, and recommendations for interpretation of the results in the clinical approach have been elaborated [24]. German researchers have suggested the utility of serial measurements of exhaled NO both at home and work for the diagnosis of OA [25]. An increase in FE_NO_ of 30–40% [26, 27] 24 h after the SIC in comparison to baseline values or an increase of 20% for baseline values > 50 parts per billion (ppb) or 10 ppb for values < 50 ppb [24] has been proposed as a significant for the diagnosis of OA. Nevertheless, the usefulness of FE_NO_ in diagnosing OA and medical certification is still controversial and less valuable than assessment of cellular IS composition due to higher sensitivity to confounding factors (e.g., infections, smoking) [22•]. In exhaled breath condensate, also 8-iso-prostaglandin has been measured as an oxidative stress biomarker in workers with OA, regardless of cellular type of airway inflammation [28].

Confirmation of non-specific bronchial hyper-responsiveness (NSBHR)-validated inhalant tests (with histamine/methacholine/mannitol/adenosine monophosphate) is indispensable part of objective diagnostic process of work-related asthma. Recording at least two measurements of NSBHR before and after the SIC may be helpful in predicting post-challenge asthmatic response in subsequent exposures (with >90% of predicted value) for the patients with OA who obtained a significant (more than two-fold) increase in NSBHR [29, 30•]. Measurements of nasal nitric oxide [31, 32] and non-specific nasal hyper-responsiveness with histamine/methacholine or cold air testing [33, 34] have been proposed as markers of local inflammation in work-related rhinitis with allergic or non-allergic background [33, 34]. However, these methods have not been validated yet, and they are not sufficiently specific [35]. Inhalant challenge with mannitol has been considered to be more specific than frequently used test with methacholine for diagnosis of asthma, including OA [36–38]. What is more, the procedure duration is shorter than in the case of applying methacholine and the costs are lower, so it may be more useful during periodical medical examinations on employees and sportsmen [39–41]. Exposure to organic dust may induce organic dust toxic syndrome (ODTS) associated with a neutrophilic response and increase in non-specific hyper-responsiveness in both the upper and lower airways [42].

In comparison to asthmatic workers, neutrophilic inflammation in IS has been observed among patients who suffered from COPD [43, 44]. What is more, biochemical analysis of IS in these samples revealed elevated levels of interleukin (IL)-6, IL-8, and tumor necrosis factor (TNF)-α. Unfortunately, there is limited data about biomarkers assessed in work-related COPD. In recently published Polish study, the evaluation of IS indicated that IL-1β, IL-6, TNF-α, and immune-reactive matrix metalloproteinase (MMP)-9 is involved in a local lower airway inflammatory process in the patients with work-related COPD in comparison to subjects with OA [18•]. The influx of neutrophilic cells into lower airways as well as elevated IL-8 level in IS were observed also in healthy subjects exposed to aluminum oxide in clinical-controlled conditions [45]. Neutrophilic inflammation with higher levels of fractional exhaled nitric oxide (FE_NO_) was found also among female hairdressers exposed to high concentrations of ammonia in comparison to unexposed controls [46]. German researchers revealed a significant positive correlation between metal concentrations and soluble inflammatory markers (total protein, MMP-9, IL-8, tissue inhibitor of metalloproteinase (TIMP)-1) in the NLF collected post-work-shift from welders exposed to chromium, nickel, manganese, and iron [47]. These findings suggest possible induction of local inflammation; however, their role in predicting the development of OR is still unclear.

## Assessment of biomarkers in skin and blood samples

Determination of skin and serum-specific immunoglobulin E (sIgE) antibodies demonstrates the sensitization to allergens; however, commercially available standardized tests are prepared only for a few occupational allergens [48, 49]. Detection of sIgE has the highest specificity of 79% in diagnosing OA induced by HMW-A and confirmed by SIC [50]. In general, sIgE detection by skin prick tests is considered to be more specific and less sensitive than serum assays in sensitized patients with airway occupational allergy [51]; however, different phenomenon has been observed in subjects with hypersensitivity to latex [52, 53]. Although, robust advantage of serum sIgE assays is the possibility to determine in patients with high risk of anaphylaxis or with disseminated skin lesions [54]. Moreover, the presence of serum sIgE has been suggested to play a role of exposure indicator to di-isocyanates in 20–50% of asthmatic subjects [55–58]. However, positive result of sIgE assay in minority of workers with isocyanate-induced asthma allowed the researchers to highlight that evaluation of serum sIgE has high specificity and low sensitivity in diagnosing occupational respiratory allergy [59, 60].

Detection of antigen-specific IgG (sIgG) antibodies is helpful in searching the causative agents of occupational hypersensitivity pneumonitis (OHP) [61, 62•], but it is not objective “gold standard” method in diagnosing OHP [63–65]. Determination of serum sIgG may be useful in predicting development of OHP [66]; however, the quantitative assessment requires different cut-off values for various agents [62•]. What is more, interpretation of the result may be difficult due to possible cross-reactivity among many fungal or bird species [67, 68]. Increased levels of serum IgG to tissue transglutaminase and serum MMP-9 have been observed in workers with toluene-di-isocyanate-induced OA [69].

Similar to IS samples, evaluation of ECP levels in serum has been considered as the exponent of systemic eosinophilic inflammation in workers with OA [70, 71]; however, this method has been not validated. Systemic inflammatory reaction characterized by increased level of TNF-α, IL-6, and IL-1β has been described as associated with ODTS [42]. Also, exposure to coal dust has been associated with elevated levels of inflammatory cytokines (TNF-α, IL-1, IL-6, IL-1β) [72]. Elevated levels of IL-1β and IL-19 have been detected in sera of patients with silicosis too [73]. Kleniewska et al. found that evaluation of serum level of C-reactive protein (CRP) can be useful in expressing systemic inflammation in patients with work-related COPD [18•] and chronically exposed to ammonia [46].

Airborne iron may be responsible for siderosis and lung cancer among welders [74, 75], and periodical quantitative assessment of serum ferritin (SF) has been proposed as a reasonable exposure biomarker in welders using high-emission technologies of respirable iron [76]. Evaluation of SF and serum transferrin levels has been also successfully applied in diagnosing methylene-di-isocyanate OA with a specificity of 85.7% [70] but not in toluene-di-isocyanate OA [77].

## Genetic predictors

Susceptibility to develop some work-related respiratory disorders has been investigated through identification of “candidate” gene researches. It has been found that the HLA-DPB11(E69) allele is closely associated with sensitivity to airborne beryllium, and inheritance of this gene increases the risk of development of chronic beryllium disease even thirty-fold in exposed workers [1, 78]. Various associations have been described between individual polymorphisms of the TNF-α gene and coal workers’ pneumoconiosis [79–81] or byssinosis [82]; however, there has not been existing any hard evidence of these interactions. Associations between gene and exposure to silicosis have been also suggested as important for polymorphisms in gene for TNF-α and for the IL-1 receptor [83–85]. Fibrosis with the background of exposure to asbestos has been considered also as a result of oxidative stress [86] and linked with the GSTM1-null genotype responsible for reducing antioxidant function [87, 88]. A few gene–work environment interactions have been found in development of di-isocyanate-induced OA (DIIA), including HLA class II genes, genes associated with the response to Th2 cytokine and with antioxidant activity, or gene related to epithelial junctional integrity [89•]. However, available data has been confusing. Some researches indicated on positive association between HLA class II allele DQB100503 and development of DIIA [90, 91] and protective effect of DQB100501 [90, 92]. On the contrary, Balboni et al. in the same study confirmed negative association between DQB100501 and DIIA [91]. The presence of HLA allele DQB100501 was considered as having protective effect on asthma development in sawmill workers exposed to western red cedar [93]. Individual susceptibility to develop DIIA was associated also with genes IL4RA, IL-13, and a CD14 promoter [94]. Polymorphism in α-T catenin gene (CTNNA3) has been strongly indicated as a potential candidate gene for DIIA susceptibility [95, 96], but the mechanism of its action on inducing and modifying the clinical course of DIIA is still unclear. It has been claimed that workers with polymorphisms in genes responsible for producing antioxidant enzymes, e.g., glutathione S-transferases (GSTs), manganese superoxide dismutase (SOD2), and microsomal epoxide hydrolase (EPHX1), may be both less [97–99] and more [100] prone to developing DIIA due to modified metabolism of di-isocyanates. Different groups of genes have been suspected of associations with OA induced by HMW-A. Polymorphisms in Toll-Like Receptors (TLRs) and CD14 may be involved in work-related airway disorders in workers exposed to endotoxin and animal allergens (laboratory and swine) [101–103] and in bakers [104, 105]. Promising group of biomarkers for allergic inflammation and rhinitis/asthma diagnosis seems to be microRNAs (miRNAs) expressed on airway epithelial cells [106, 107]; however, any published data about these assays in work-related airway disease has not been found.

## Conclusions

At present, most of the well-known and reliable biomarkers are related to work-related airway disease with allergic background. Numerous researches have been carried out on occupational asthma and resulted in indicating the cellular assessment of induced sputum as the most useful in diagnostic process. A comprehensive overview of current knowledge on biomarkers’ utility in diagnosis of occupational asthma was described elsewhere [108•]. Biomolecular investigations on association between human genome and workplace inhalant exposure need to be wider explored in the future. Identifying “at-high-risk” job applicants may be the most successful intervention in primary prophylaxis of work-related disorders.
